# Timing of social distancing policies and COVID-19 mortality: county-level evidence from the U.S.

**DOI:** 10.1007/s00148-021-00845-2

**Published:** 2021-04-08

**Authors:** Catalina Amuedo-Dorantes, Neeraj Kaushal, Ashley N. Muchow

**Affiliations:** 1 Economics and Business Management, University of California Merced, Merced, CA USA; 2 School of Social Work, Columbia University, New York, NY USA; 3 Criminology, Law, and Justice, University of Illinois at Chicago, Chicago, IL USA

**Keywords:** COVID-19, Mortality, Infections, Non-pharmaceutical interventions, United States, I1, I10, I18

## Abstract

Using county-level data on COVID-19 mortality and infections, along with county-level information on the adoption of non-pharmaceutical interventions (NPIs), we examine how the speed of NPI adoption affected COVID-19 mortality in the United States. Our estimates suggest that adopting safer-at-home orders or non-essential business closures 1 day before infections double can curtail the COVID-19 death rate by 1.9%. This finding proves robust to alternative measures of NPI adoption speed, model specifications that control for testing, other NPIs, and mobility and across various samples (national, the Northeast, excluding New York, and excluding the Northeast). We also find that the adoption speed of NPIs is associated with lower infections and is unrelated to non-COVID deaths, suggesting these measures slowed contagion. Finally, NPI adoption speed appears to have been less effective in Republican counties, suggesting that political ideology might have compromised their efficacy.

## Introduction

The COVID-19 pandemic and non-pharmaceutical interventions (NPIs) implemented in many countries to suppress contagion have unsettled lives fundamentally and cratered the global economy. Epidemiologists contend that NPIs (i.e., safer-at-home orders, closures of non-essential businesses and schools, or bans on large gatherings) combined with testing, tracing, and isolating are the only options to fight the pandemic until a vaccine is widely available or societies achieve widespread immunity (Ferguson et al. 2006; Karlsson et al. 2014; Tian et al. 2020). Yet, the intensity and vigor of NPI implementation have varied across countries, reflecting skepticism regarding their efficacy and concerns about their social and economic costs.

In the United States, where COVID-19 has taken a high toll in terms of infections and mortality, skepticism toward NPIs reigns high among the public and legislators. Early in the pandemic, President Trump famously criticized NPIs by remarking that “the cure cannot be worse than the problem itself” (Haberman and Sanger 2020). The nation remained divided on the effectiveness of NPIs, even as the pandemic raged from March to early May 2020 in the Northeast, spreading more widely to the rest of the country thereafter. Surveys show that conservative Republicans expressed more skepticism than liberal Democrats about NPIs (Funk and Tyson 2020). State and local implementation and lifting of NPIs were often driven by political ideology. Republican-governed cities were slower to adopt NPIs, whereas cities led by Democrats were more aggressive (Willetts 2020).

Amid the highly partisan response to the pandemic, the question remains: does the timeliness of NPIs save lives in the United States? Have these interventions helped reduce the spread of the virus? Has political ambivalence toward NPIs influenced their effectiveness? We address these questions in this paper using county-level data on mortality, infections, and NPIs.

If NPIs have not been successful in the United States, that would mean the government has needlessly cratered the economy, compromised children’s education, disrupted lives and livelihoods, and reduced the pace at which herd immunity can be achieved—ultimately validating public skepticism about these policies. Arguably, NPIs reduce the pace at which a population can acquire widespread immunity. For this reason, several countries, including the United Kingdom in the initial stages of the pandemic and Sweden, opted against implementing NPIs. Additionally, the implementation of NPIs inevitably brings economies to a halt, resulting in tidal unemployment claims. Many countries and localities delayed their adoption and effective implementation to lessen their economic and social toll. These delays could have adversely affected the spread of the pandemic. Indeed, if NPIs are effective at reducing contagion, the politicization of NPIs can be blamed for the ambivalence and hesitation toward their implementation. This ambivalence and hesitation could explain the United States’ failure to contain the virus, even as other developed countries have successfully reduced infections and mortality.

A rapidly growing body of literature has examined the impact of NPIs on COVID-19 infections and deaths using Chinese data (Pan et al. 2020; Qiu et al. 2020), Spanish data (Amuedo-Dorantes et al. 2020), and cross-national data (e.g., Anderson et al. 2020; Bai et al. 2020; Flaxman et al. 2020; Hsiang et al. 2020; Imai et al. 2020; Viner et al. 2020). Focusing on the United States, several researchers have explored the role of various social distancing measures on the incidence of COVID-19 and COVID-related mortality. For instance, Auger et al. (2020) focus on school closures, Korevaar et al. (2020) use a low/medium/high NPI index, VoPham et al. (2020) use smartphone GPS data to estimate social distancing, and Wright et al. (2020) focus on the role of mask mandates.^1^ Other studies, more closely related to ours, have focused on the role of safer-at-home orders (i.e., Dave et al. 2020; Fowler et al. 2020; Friedson et al. 2020; Harris 2020b).^2^ These studies find that NPIs are associated with lower infection and mortality rates—some focused in California or New York (i.e., Friedson et al. 2020; Harris 2020b) and others countrywide (*i.e.*, Dave et al. 2020; Fowler et al. 2020). We build on this research by assessing the relevance of the timing of two of the most frequently adopted NPIs—safer-at-home orders and non-essential business closures—on mortality. Our paper makes two primary contributions. First, we study the effectiveness NPIs in relation to their timeliness. To that end, we construct a measure that captures the relative speed of NPI adoption based on a county’s rate of contagion when the NPI was adopted.^3^ Using this measure of timeliness, as opposed to just the policy adoption date, is critical from an epidemiological point of view, as what is considered “early” implementation in some localities might be “late” for others depending on their position on the pandemic curve. Second, we investigate the mechanism through which NPIs impacted COVID deaths. Specifically, we investigate whether NPIs saved lives by curtailing the spread of infection or by reducing pressure on the healthcare system.

Further, we investigate whether NPI efficacy differed across counties with different political ideologies and different degrees of demographic, economic, and health-related vulnerabilities. To examine the former, we construct a dummy to identify Republican counties, defined as those where most residents voted for President Trump in the 2016 election, and estimate whether NPI efficacy differed in those areas compared to other counties. For the latter, we use several pre-COVID demographic, economic, and health characteristics to explore the differential efficacy of NPIs across counties with distinct degrees of vulnerability. Ideally, we would use time series data on COVID-19 mortality according to these traits, but such data are currently not available. Instead, we use pre-COVID county-level characteristics to explore differences in the relevance of NPI adoption timing across counties with different characteristics associated with poor COVID-19 health outcomes. Finally, we explore mechanisms through which NPI adoption speed might be critical, focusing on the spread of the infection and the ability to avert an overwhelmed healthcare system.

A challenge in estimating the causal effect of NPIs on mortality is that these interventions are adopted in response to the spread and severity of the virus. Because of the likely presence of reverse causality (i.e., COVID-linked deaths motivating the adoption of NPIs), a simple correlation between NPIs and COVID-linked mortality or infection will likely result in downwardly biased estimates. We address this concern by supplementing our primary analysis with an event study examining how COVID-19 mortality rates respond to NPI adoption.

Because of the ongoing nature of the pandemic, an additional challenge is the chosen temporal frame for our analysis. We focus on the early months of the pandemic, capturing when states and counties first adopted NPIs, through the first re-opening. This means we are comparing counties at various initial stages of the pandemic. To address this limitation, we estimate models that separate specific outliers during that period. Specifically, we experiment with samples that include only Northeastern states—which comprised the epicenter of the pandemic during our study period; exclude Northeastern states; or exclude the state of New York. These sample modifications allow us to compare NPI speed between counties in roughly similar stages of the pandemic.

Any research on the efficacy of NPIs in the United States is affected by the fact that data on reported infections and COVID-linked mortality are highly correlated with COVID-19 testing, which has varied across the country and over time. In counties with inadequate testing, reported infections likely underestimate actual infections and deaths attributable to COVID-19 are likely to be reported as non-COVID mortalities. Furthermore, if testing is correlated with NPIs, it will confound the estimated efficacy of NPIs. To address these concerns, we explicitly control for testing. Similarly, to address the possibility that our NPI estimates reflect endogenous self-distancing occurring before NPI adoption, we include robustness checks that control for the daily median maximum distance traveled by county residents as an estimate of mobility at the county level.

To explore the underlying mechanisms at play, we examine how NPI adoption speed affects infections and conduct state-level analyses of the association between NPIs and non-COVID deaths.^4^ Studies document that non-COVID deaths increased over our study period (Woolf et al. 2020). This could have occurred for various reasons, including the voluntary postponement of procedures or, in some instances, through an overwhelmed healthcare system. If timely adoption of the NPIs helped reduce the burden on the healthcare system, they should also lower non-COVID deaths.

We find that advancing the implementation date of NPIs by 1 day before the doubling of infections would have lowered the COVID-19 death rate by 1.9%. The value of early policy implementation proves robust to the use of alternative measures of NPI adoption speed; to controlling for testing, other NPIs, and mobility; as well as to the removal of outliers (i.e., New York and the Northeast region) from the analysis. We find that NPI adoption speed is associated with fewer infections, suggesting these measures operated by slowing contagion. We also find that NPI adoption speed is not associated with fewer non-COVID deaths, which suggests that the identified effectiveness of NPIs in reducing COVID mortality are not spurious. Finally, our results suggest that the speed of NPI adoption proves less effective in Republican counties, suggesting that the attitudes of residents toward NPIs may influence their efficacy.

## Data

### Mortality and infections

To determine how NPIs affected COVID-19 mortality in the United States, we use county-level data on COVID-19 deaths and infections collected by Johns Hopkins University. This data includes daily counts of cumulative COVID-19 cases and deaths reported by state and local health departments.^5^ We use 2019 population estimates from the U.S. Census Bureau to derive daily COVID-19 mortality rates by county.

We focus our analysis on the period from February 15, 2020, to April 23, 2020. While the first confirmed case of COVID-19 occurred in late January, countrywide contagion was reported in late February and early March. To avoid confounding the effects of NPI adoption speed with the continuation of NPI policies, our study period ends on April 23, 2020—the day before the first NPI was lifted.^6^

We also collect information on state-level testing and overall mortality from the COVID Tracking Project and the Centers for Disease Control and Prevention, respectively. Given that the identification of COVID-19 infections and cause of death attributions are contingent upon detection, we use the most detailed information available from the COVID Tracking Project—daily test results by state—to account for variation in testing.^7^ We also collect information on mortality by cause of death from the Centers for Disease Control and Prevention to estimate non-COVID deaths.^8^ We use the most detailed information available—weekly deaths by state—to explore possible mechanisms responsible for any observed impact of NPIs on COVID-19 mortality. While county-level information would be preferable to prevent any information loss, the state level data allows us to account for differences in the level testing at the state level, as well as modeling non-COVID deaths to learn about potential mechanisms at play at the state level.

### Non-pharmaceutical interventions

We use data from the National Association of Counties (NACo) and Boston University’s School of Public Health to identify counties with NPIs in place during our study period. The NACo compiles information on the type and timing of NPIs for every county in the United States.^9^ We complement this information with a comprehensive database assembled by Boston University researchers that records similar measures taken by states.^10^ We focus on two types of NPIs: non-essential business closures and safer-at-home policies.

While there is overlap between non-essential business closures and safer-at-home orders, business closure policies only restrict the activities of certain businesses, whereas safer-at-home orders include provisions that close non-essential businesses in addition to restricting individual movement. Safer-at-home policies—also referred to as “stay-at-home” and “shelter-in-place”—explicitly restrict the movement and activities of individual residents unless they are engaged in “essential” activities.^11^ These policies prohibit residents from gathering or travelling outside of their homes unless for an essential activity and, as such, often consist of the closure of non-essential businesses (i.e., restaurants, bars, gyms). Though policies and enforcement vary, residents who ignore safer-at-home orders may face a misdemeanor punishable by fine, imprisonment, or both (Harris 2020a).

We also use information manually curated by a dedicated group of Virtual Student Federal Service Interns to identify the type and timing of other NPIs commonly adopted over our study period.^12^ Using this data, we identified whether and when a state or county adopted any of the following NPIs: (1) mandated face mask use by individuals in public spaces; (2) K-12 school closures; (3) nursing home visitation restrictions; (4) gym closures; and (5) dine-in restaurant closures.

### Mobility

We make use of daily mobility data for each county obtained from Descartes Labs to account for variation in compliance with the social distancing imposed by the NPIs.^13^ Commercially available location data from smartphones and other mobile devices are used to sample the movement of individuals (Warren and Skillman 2020). We use estimates of the median maximum distance traveled by residents to estimate daily mobility at the county level. These statistics are available from March 1, 2020, and beyond.

### County characteristics

To investigate whether NPI efficacy differed across counties with different political ideologies and different degrees of demographic, economic, and health-related vulnerabilities, we collect information on a series of county-level characteristics. We use data from the MIT Election Lab on the share of residents that voted Republican in the 2016 presidential election to identify majority-Republican counties—defined as those with a Republican vote share of 50% or more.^14^ We collect information on a series of demographic and socioeconomic characteristics sourced from 2018 5-year American Community Survey. Specifically, we compile estimates on the percent of county residents that are (i) over the age of 65, (ii) without health insurance, (iii) unemployed, and (iv) living below the federal poverty line. We use data from the Centers for Medicare and Medicaid Services to measure county-level chronic disease prevalence. We use information from 2017, the latest year available, to create a comorbidity index that aggregates the percent of Medicare beneficiaries with chronic diseases associated with severe COVID-19 outcomes, including chronic lung disease (chronic obstructive pulmonary disease, asthma), heart conditions (atrial fibrillation, heart failure, ischemic heart disease), cancer, hypertension, HIV/AIDS, diabetes, chronic kidney disease, and liver disease (hepatitis).^15^ We standardize the index to have a mean of 0 and a standard deviation of 1, with larger values indicating higher comorbidities. Lastly, we combined 2019 population estimates with data from the U.S. Census Bureau on county land area to measure population density as the number of persons per square mile.^16^

## Methodology

Our primary objective is to explore the effectiveness of NPIs on COVID-19 mortality. To that end, we start by estimating the following baseline difference-in-differences specification:

1 $$ {Y}_{\mathrm{c}\mathrm{t}}=\alpha +\beta \left({Post}_{\mathrm{c}\mathrm{t}}\times NPI\ {speed}_{\mathrm{c}}\right)+{\rho}_{\mathrm{c}}+{\vartheta}_{\mathrm{t}}+{\varepsilon}_{\mathrm{c}\mathrm{t}} $$

where the vector *Y*_ct_ represents the number of COVID deaths per 100,000 in county *c* and date *t*.

We consider two different types of NPIs: safer-at-home policies and non-essential business closures. Our main regressor is an interaction term made up of two variables: (1) *Post*_ct_, a dummy variable indicative of the period in county *c* when the NPI was in effect, and (2) *NPI speed*_c_, the relative speed of NPI adoption based on county *c*’s rate of contagion when the NPI was adopted. The post-NPI dummy (*Post*_*ct*_) includes a 14-day lag to account for the average amount of time between infection and possible death (Lauer et al. 2020). The vector *NPI speed*_c_ measures the number of days between the adoption of the NPI and the first day-to-day doubling of county-level infections per capita.^17^ We multiply this count by minus one, so that higher values indicate a faster response.^18^ This operationalization allows us to gauge the impact of the adoption of NPIs as well as the effect of a faster response. We contrast two counties in the state of New York to illustrate. The New York City boroughs were late adopters—evidenced in their (-15) day NPI speed measure. New York’s statewide safer-at-home order and non-essential business closure was adopted on March 22—15 days *after* New York City saw its first day-to-day doubling of infections per capita on March 7. Saratoga County, on the other hand, did not experience their first day-to-day infection doubling until March 24—17 days *after* statewide NPIs were adopted—making this county an early adopter, evidenced in their 17-day NPI speed measure.

Equation (1) also includes daily fixed effects to capture temporal shifts in the incidence and treatment of the disease across the country and county fixed effects to account for time-invariant differences potentially related to COVID mortality, such as population density, health infrastructure, and population comorbidities. Standard errors are clustered at the county level.

## Descriptive evidence

**Fig. 1:**
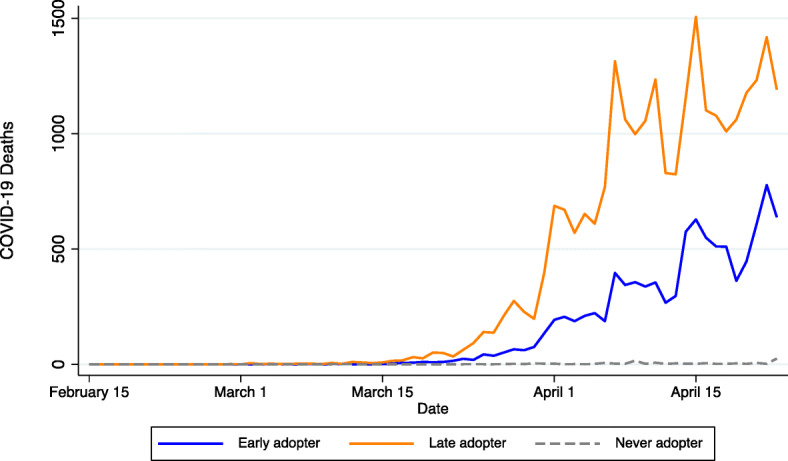
Daily COVID-19 mortality by non-pharmaceutical intervention timing

**Fig. 2:**
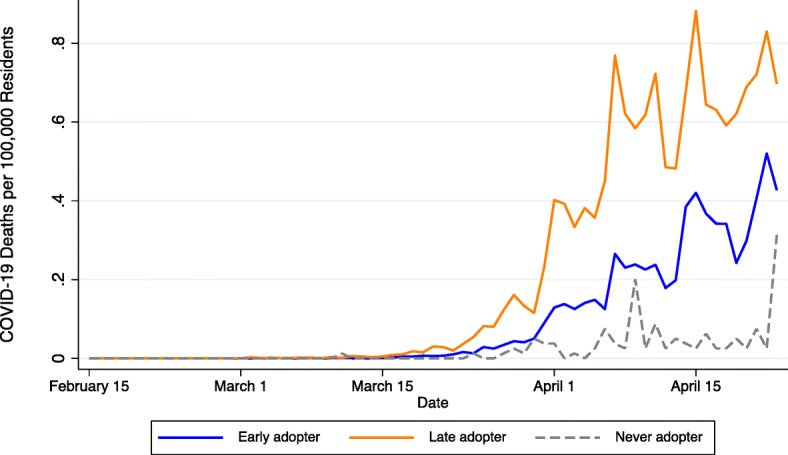
Daily COVID-19 mortality rates by non-pharmaceutical intervention timing. Early adopters include counties with safer-at-home orders or non-essential business closures in place before the first day-to-day doubling of infections per capita. Late adopters include counties that adopted an NPI after the first day-to-day infection doubling. Never adopters include counties that did not have a safer-at-home order or non-essential business closure in place anytime between February 15, 2020, and April 23, 2020

Our methodological approach is inspired by the daily variation in COVID-19 deaths and deaths per capita displayed in Figs. 1 and 2, respectively. As shown therein, early versus late adopters of NPIs were impacted differently. We distinguish among three types of counties: (1) early adopters, which include those with a safer-at-home order or business closure in place *prior to* the first day-to-day doubling of infections per capita; (2) late adopters, which include counties that adopted an NPI *after* the first day-to-day doubling; and (3) counties that *never* adopted a safer-at-home order or business closure policy during our study period. COVID-19 deaths began to accelerate in mid-March for early and late adopters but at notably different rates. Peak COVID-19 death rates in counties classified as late adopters were more than 2.5 times the peak experienced by early adopters. Also noteworthy is the relative dearth of COVID-19 mortality in the 311 counties that were not subject to an NPI during our study period. Most of these counties are in sparsely populated states (i.e., North and South Dakota, Nebraska, and Wyoming).

**Fig. 3:**
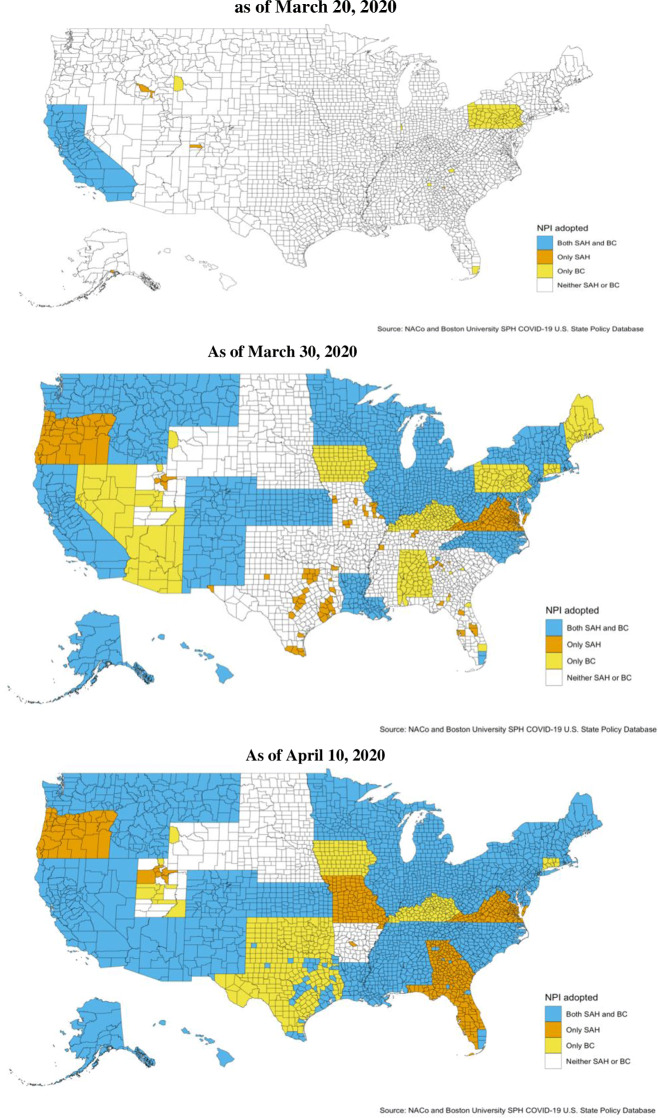
Geographic variation in adoption of non-pharmaceutical interventions

Figure 3 illustrates the staggered adoption of safer-at-home and non-essential business closure policies, which provides the temporal and geographic variation needed for identification. The first statewide NPI was adopted by California on March 19, 2020. By March 20, a total of 134 counties, including California’s 58, had an NPI in place. As illustrated in the subsequent maps, most NPI adoptions occurred in late March and early April. By March 30, a total of 1979 counties were subject to a safer-at-home order, non-essential business closure, or both. This number grew to 2806 by April 6—after which, according to our sources, no additional safer-at-home or non-essential business closures were implemented during the period covered in this study.

Table 1 displays basic descriptive statistics for the full sample and by NPI adoption timing. Counties were observed daily from February 15, 2020, to April 23, 2020 (69 days). The descriptive statistics confirm the trends illustrated in the figures. COVID-19 infections and mortality were lower in early-adopting counties when compared to counties adopting NPI measures late, despite the larger number of tests performed in the latter group. Counties that never adopted a safer-at-home order or non-essential business closure were the smallest in size and had COVID-19 infections and mortality figures that were well below those of late and early NPI adopters. There are other differences across counties worth noting. For instance, late-adopting counties were, by far, the largest in population size and density. Early-adopting counties were somewhat between never adopters and late adopters in terms of size; they were also more mobile and quicker to adopt other NPI before infections doubled. In terms of pre-COVID characteristics, early-adopting counties had a larger share of elderly residents, had more residents living in poverty, and were more likely to be classified as Republican than late-adopting counties. Counties that never adopted NPIs were more likely to have health insurance and have lower levels of unemployment, poverty, and comorbidity indexes relative to counties that adopted NPIs early. Differences in unemployment rates, health insurance, and comorbidity indexes were not different across counties that adopted NPIs early or late.

**Table 1:** Descriptive statistics

Sample	Overall		By NPI adoption timing					
			Early adopters		Late adopters		Never adopters	
Variable	Mean	SD	Mean	SD	Mean	SD	Mean	SD
COVID deaths per 100,000	0.07	0.77	0.06	0.74	0.20*	1.12	0.01*	0.41
COVID infections per 100,000	1.65	11.77	1.35	10.53	4.09*	12.38	1.11*	18.18
Population	105,306	359,430	61,180	240,035	469,397*	751,770	25,649*	64,489
Population per square mile	224.66	944.78	134.45	577.45	993.26*	2171.31	32.54*	115.36
NPI speed	31.00	40.08	36.55	40.08	−6.21*	4.14	NA	NA
Safer-at-home speed	26.14	38.24	32.12	38.51	−7.40*	4.78	NA	NA
Non-essential business closure speed	31.65	40.65	36.57	40.62	−6.61*	4.25	NA	NA
Other NPI speed	38.98	41.94	44.82	42.38	5.24*	5.76	32.66*	43.02
State test results per 100,000	298.88	471.29	289.18	453.75	348.86*	575.77	316.49*	466.43
Mobility index	6.12	15.27	6.49	16.92	4.20*	3.96	5.79*	6.54
Majority Republican (2016)	0.81	0.40	0.84	0.37	0.48*	0.50	0.93*	0.25
Percent over age 65 (2018)	18.38	4.59	18.63	4.34	15.69*	4.87	19.57*	4.98
Percent without health insurance (2018)	8.21	3.99	8.28	4.05	8.45	3.53	7.32*	3.93
Percent unemployed (2018)	1.22	0.59	1.26	0.58	1.32	0.44	0.77*	0.56
Percent living below FPL (2018)	10.74	4.17	10.99	4.14	9.92*	3.83	9.76*	4.55
Comorbidity index (2017)	0.00	1.00	0.06	1.01	0.12	0.86	−0.57*	0.84
Observations	215,073		168,498		25,116		21,459	

Notes: Statistics are reported at the county level unless otherwise specified. These estimates are not weighted by population. Counties that never adopted an NPI during our study period were assigned an uninformative NPI speed value to ensure these cases were preserved when estimating the model outlined in Eq. 1. Our specification interacts NPI speed with a dummy indicative of the day a county adopted an NPI—effectively rendering this value equal to zero for never adopters. *p<0.05 in *t*-test comparing value with early adopters

## Main findings and robustness checks

Our main objective is to learn about the importance of timing when adopting NPIs in fighting COVID-19. If timing proves critical, a secondary objective is to gain a better understanding of the channels enabling the effectiveness of NPIs in curtailing deaths—an investigation that involves uncovering heterogeneous impacts of the adopted NPIs.

To achieve our primary aim, we start by estimating several model specifications of Eq. (1). Initially, we simply consider the adoption timing of either safer-at-home orders or non-essential business closures—the most common NPIs during the pandemic. The results from this exercise are displayed in Table 2. The baseline specification only includes date and county fixed effects. Subsequently, we control for the level of testing in the state, the speed of adopting other NPIs, as well as mobility measured as the median maximum distance traveled by county residents. COVID *testing* was inadequate in the initial months after the outbreak but began to improve over our study period. To account for any mechanical association between testing, infections, NPIs, and COVID mortality, we control for daily trends in state-level testing.^19^ Accounting for the speed of adopting *other NPIs*—including mask mandates, school and gym closures, nursing home visitation restrictions, and dine-in restaurant closures—prevents us from confounding the effects of safer-at-home orders and non-essential business closures with other NPIs. This other NPI speed measure reflects the number of days between the earliest date any of the other NPIs were adopted and the first day infections per capita doubled. Controlling for *mobility* allows us to capture the role played by any endogenous self-distancing irrespective of whether the NPIs were in place. Regardless of the model specification used, the speed of adoption of NPIs curtails COVID-19 deaths.^20^ Based on the estimates from our preferred model specification that controls for testing and the speed of adopting other NPIs (column (3) in Table 2), we find that moving up the implementation date of any of the NPIs (if both were adopted, whichever came first) by 1 day lowers the COVID-19 death rate by 1.9%.^21^

**Table 2:** Impact of NPI speed on COVID-19 deaths per 100,000 residents

Model specification	Baseline	Control for testing	Control for other NPI speed	Control for mobility
Column	(1)	(2)	(3)	(4)
*Post*_*ct*_ × *NPI speed*_*c*_	−0.0019***	−0.0020***	−0.0013***	−0.0013***
	(0.0002)	(0.0002)	(0.0002)	(0.0002)
State test results per 100,000		0.0003***	0.0003***	0.0003***
		(0.0000)	(0.0000)	(0.0000)
*Post*_*ct*_ × *Other NPI speed*_*c*_			−0.0012***	−0.0018***
			(0.0001)	(0.0001)
Mobility				−0.000001
				(0.00005)
Observations	215,073	215,073	215,073	141,480
*R*-squared	0.083	0.088	0.089	0.127
Dependent variable mean	0.070	0.070	0.070	0.100

Notes: *** *p*<0.01, ** *p*<0.05, * *p*<0.1. All regressions include a constant term, date, and county fixed effects. Standard errors are in parentheses and clustered at the county level. This table reports the estimates from Eq. (1) using daily COVID-19 deaths occurring between February 15, 2020, and April 23, 2020. Column (2) controls for state-level testing, column (3) further controls for the speed of adopting other NPIs, and column (4) controls for residential mobility. The estimates reported in column (4) use daily COVID-19 deaths for 2260 counties with mobility data for the period March 1, 2020, to April 23, 2020. We re-estimated the models presented in columns (1) to (3) using this restricted sample, as shown in Panel A of Table 8 in the Appendix. While our estimates increase in magnitude in the first two columns, the estimates from our preferred specification in column (3) are nearly identical

To gain a better understanding of which of the two most common NPIs matter the most, we re-estimate the models including separate measures of adoption speed for each NPI. As can be seen in Table 3, our estimates suggest both measures prevent deaths. Estimates from our preferred specification in column (3) reveal that adopting non-essential business closures 1 day before infections double lowers COVID-19 deaths by 0.7% and moving up the adoption of a safer-at-home order by 1 day curtails COVID-19 mortality rates by 1.3%.^22^ Recognizing the overlap between these two NPIs, we experimented with adding an interaction term to assess whether the effect of one policy is absorbed by the other. Results are presented in Panel C of Table 8 in the Appendix. While the two NPIs prove individually effective with their impact rising when implemented jointly in models (1) and (2), their individual effectiveness dissipates as we further control for the speed of adopting other NPIs, possibly owing to correlations among all NPIs.

**Table 3:** Impact of disaggregated NPI speeds on COVID-19 deaths per 100,000 residents

Model specification	Baseline	Control for testing	Control for other NPI speed	Control for mobility
Column	(1)	(2)	(3)	(4)
*Post*_*ct*_ × *BC Speed*_*c*_	−0.0016***	−0.0011***	−0.0005***	−0.0007***
	(0.0002)	(0.0002)	(0.0002)	(0.0002)
*Post*_*ct*_ × *SAH Speed*_*c*_	−0.0008***	−0.0011***	−0.0009***	−0.0009***
	(0.0002)	(0.0002)	(0.0002)	(0.0002)
State test results per 100,000		0.0003***	0.0003***	0.0003***
		(0.0000)	(0.0000)	(0.0000)
*Post*_*ct*_ × *Other NPI speed*_*c*_			−0.0011***	−0.0016***
			(0.0001)	(0.0001)
Mobility				−0.000001
				(0.00004)
Observations	215,073	215,073	215,073	141,480
*R*-squared	0.083	0.088	0.089	0.127
Dependent variable mean	0.070	0.070	0.070	0.100

Notes: *** *p*<0.01, ** *p*<0.05, * *p*<0.1. All regressions include a constant term, date, and county fixed effects. Standard errors are in parentheses and clustered at the county level. This table reports the estimates from Eq. (1) using daily COVID-19 deaths occurring between February 15, 2020, and April 23, 2020. Column (2) controls for state-level testing, column (3) further controls for the speed of adopting other NPIs, and column (4) controls for residential mobility. The estimates reported in column (4) use daily COVID-19 deaths for 2260 counties with mobility data for the period March 1, 2020, to April 23, 2020. We re-estimated the models presented in columns (1) to (3) using this restricted sample, as shown in Panel B of Table 8 in the Appendix. While our estimates increase in magnitude in the first two columns, the estimates from our preferred specification in column (3) are nearly identical

Finally, in Table 4, we conduct several robustness checks to assess the sensitivity of our findings to: (1) alternative measures of the NPI adoption speed; (2) the application of population weights to derive nationally representative estimates; and (3) using different samples that exclude New York and the Northeast region as potential outliers and isolate the Northeast region.^23^ In what follows, we briefly refer to each robustness check.

**Table 4:** Robustness checks—impact of NPI speed on COVID-19 deaths per 100,000 residents

Robustness check	Alternative contagion threshold		Alternative weighting	Alternative samples		
Column	(1)	(2)	(3)	(4)	(5)	(6)
Model specification	Pre-NPI national average	Pre-NPI county average	Population weighted	Excluding NY	Excluding NE region	Only the NE region
*Post*_*ct*_ × *NPI speed*_*c*_	−0.0012***	−0.0013***	−0.0035***	−0.0012***	−0.0009***	−0.0099***
	(0.0002)	(0.0003)	(0.0012)	(0.0002)	(0.0001)	(0.0021)
State-level tests per 100,000	0.0003***	0.0003***	0.0006***	0.0003***	0.0002***	0.0001*
	(0.0000)	(0.0000)	(0.0001)	(0.0000)	(0.0000)	(0.0001)
*Post*_*ct*_ × *Other NPI speed*_*c*_	−0.0007***	−0.0030***	−0.0030***	−0.0012***	−0.0010***	−0.0064***
	(0.0001)	(0.0003)	(0.0004)	(0.0001)	(0.0001)	(0.0013)
Observations	215,073	215,073	215,073	211,071	200,721	14,352
*R*-squared	0.088	0.089	0.274	0.084	0.072	0.251
Dependent variable mean	0.070	0.070	0.155	0.068	0.060	0.207

Notes: *** *p*<0.01, ** *p*<0.05, * *p*<0.1. All regressions include a constant term, date, and county fixed effects. This table reports estimates using specification (3) from Table 2 that predicts daily COVID-19 deaths occurring between February 15, 2020, and April 23, 2020. In columns (1) and (2), we alter the definition of contagion we used to measure the speed of NPI adoption. Specifically, we replace our original contagion threshold, which reflected the first day-to-day doubling of infections per capita in a given county to (1) the first day infections per capita exceeded the national average from January 21, 2020, to March 7, 2020, and (2) the first day infections per capita exceeded the overall county average prior to any NPI adoption, the results of which are found in columns (1) and (2), respectively. In column (3), we apply population weights to derive nationally representative estimates. In columns (4), (5), and (6), we experiment with using alternative samples. In column (4), we exclude New York from the analysis. In column (5), we exclude the entire Northeast region, which consists of Connecticut, Maine, Massachusetts, New Hampshire, Rhode Island, Vermont, New Jersey, New York, and Pennsylvania. In column (6), we focus exclusively on the Northeast region

As noted earlier, the estimates in Table 2 use a contagion threshold equal to the first day-to-day doubling of infections per capita in each county. In columns (1) and (2) of Table 4, we experiment with different contagion thresholds: the first day infections per capita exceeded the national average from January 21, 2020, to March 7, 2020 (column 1), and the first day infections per capita exceeded the county average prior to any NPI adoption (column 2).^24^ Our results prove robust to the use of these alternative contagion thresholds. Accelerating the adoption speed of the NPIs by 1 day lowers the COVID-19 mortality rate by 1.7% in column (1) and by 1.9% in column (2).

Next, we experiment with using population weights to derive nationally representative estimates. As can be seen in column (3) of Table 4, we continue to find that speeding up the adoption of the NPIs by 1 day before infections double would have significantly lowered mortality from COVID-19, in this case by 2.3%, as opposed to 1.9% using the unweighted estimates of Table 2—which may suggest that NPIs are more effective in more populous counties, where the transmissibility of the virus is greater.

Finally, we test the sensitivity of our findings across different geographic samples. First, we exclude New York—which was the epicenter of the pandemic during the period under consideration—to check if our results were driven by its presence in the sample. As can be seen in column (4) of Table 4, our results remain robust to this exclusion. Speeding up NPI adoption by 1 day before infections double would have lowered the COVID-19 mortality rate by 1.8% if we exclude New York. We next experiment with excluding the entire Northeast region (column 5), as well as focusing entirely on that region (column 6). As shown therein, the results confirm our prior findings, underscoring the significance of NPIs in lowering mortality in the Northeast. Specifically, speeding up the implementation of the NPIs by 1 day would have lowered COVID-19 deaths by 1.5% if we exclude the entire Northeast region. However, in that region alone, deaths from COVID-19 would have dropped by 4.8%.

In sum, the analyses in Table 4 confirm the robustness of our estimates presented in Table 2 to alternative definitions of contagion, to the use of population weights, and to changes in the geographic scope of our sample.^25^

## Identification

An important caveat of the difference-in-differences approach adopted above is the non-random adoption of NPIs. Given their implicit economic cost, counties are likely reticent to impose social distancing, unless it is suspected that the healthcare system will be overwhelmed as the death toll climbs. Fortunately, from an inferential standpoint, if NPIs are implemented once contagion has surpassed a threshold, the estimated impact of NPIs in curtailing COVID-related deaths would likely represent a lower bound estimate of the true effectiveness of the adopted measures if they were adopted in a timely manner. A related concern refers to endogeneity biases stemming from unobserved heterogeneity. For instance, if the adoption of an NPI is related to unobserved or unaccounted for factors, such as the county’s political ideology—which may affect timely adoption of NPIs as well as cause laxity in the adoption of other measures (Dave et al. 2020)—the estimated impact of NPIs might be confounded by other unobserved factors.

To address these endogeneity concerns, we conduct an event study. Such an approach allows us to gauge if COVID-19 mortality trends systematically differed across counties before the adoption of NPIs. In this manner, we first address concerns regarding differential pre-trends across early- versus late-adopting counties. Secondly, we determine whether there is a clear break in the trend of COVID-19 mortality following the adoption of NPIs. This enables us to dissipate concerns regarding the confounding impact of unobserved and unaccounted for factors. Finally, we examine the dynamic impact of NPIs and assess the plausibility of how these policies reduce infections and deaths. Specifically, we estimate the following model:

2 $$ {Y}_{ct}=\alpha +\sum \limits_{-35}^{35}{\beta}_t{Post}_{ct}+{\rho}_c+{\vartheta}_t+{\varepsilon}_{ct}. $$

Equation (2) differs from Eq. (1) in that it includes leads and lags to NPI adoption, allowing us to examine the existence of pre-trends up to 35 days prior, as well as dummies for up to 35 days after NPI adoption to learn about the impact of the implemented policies. A developing literature (e.g., de Chaisemartin and D'Haultfœuille 2018, 2020; Callaway and Sant'Anna 2020; Goodman-Bacon 2018) points to challenges in interpreting estimates from difference-in-differences models when treatment effects are heterogeneous across time. Specifically, Goodman-Bacon (2018) shows how treatment effects that take place in a staggered fashion can result in difference-in-differences estimates that are biased away from a weighted mean of the average treatment effect on the treated. Our focus on a relatively short span of time should minimize concerns surrounding staggered treatment effects. However, to homogenize treatment effects, we also experiment with excluding early-adopting counties from the event study.

**Fig. 4:**
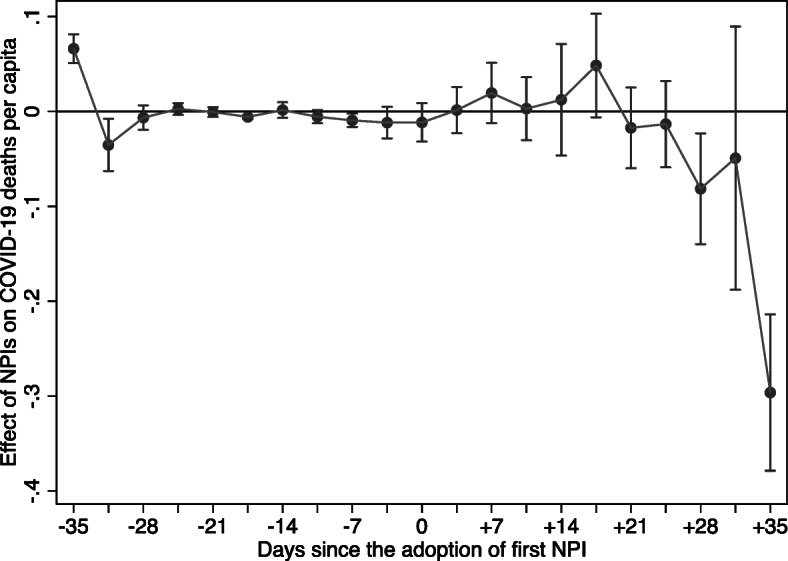
Event study non-pharmaceutical invention effects on COVID-19 deaths per capita. This figure plots the β_t coefficients from Eq. 2, including controls for state-level testing, other NPI adoption speed, and residential mobility. Bands represent 95% confidence intervals for each estimate

**Fig. 5:**
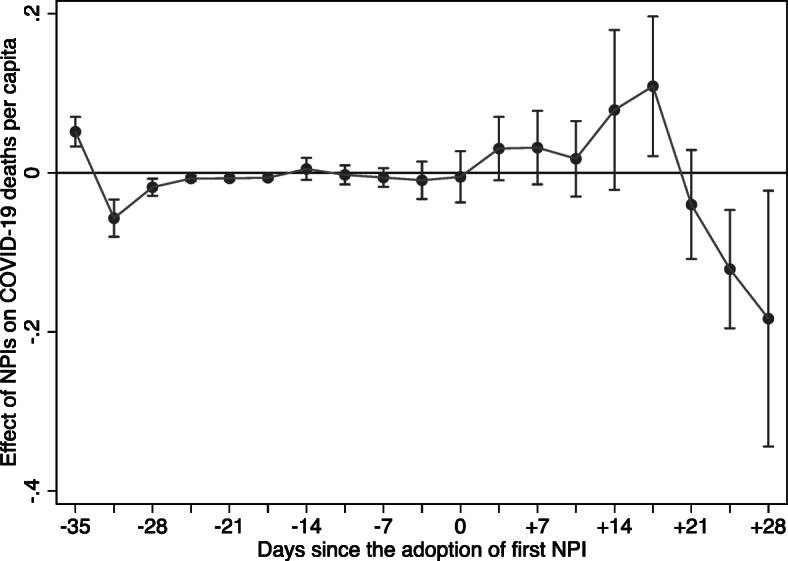
Event study excluding counties implementing NPIs prior to March 26, 2020, (non-pharmaceutical intervention effects on COVID-19 deaths per capita). This figure plots the β_t coefficients from Eq. 2, including controls for state-level testing, other NPI adoption speed, and residential mobility. Bands represent 95% confidence intervals for each estimate

Figure 4 displays the coefficients from the event study corresponding to the model specification presented in column (4) of Table 2, along with 95% confidence intervals. All estimates for the days preceding the adoption of the first NPI in the county are close to zero, strongly supporting the assumption of no differential pre-trends. In addition, there is a clear break in the trend of COVID-19 deaths approximately 4 weeks after NPI adoption—a common turn around period from infection to recovery for most mild cases (e.g., Britt 2020; Zhou et al. 2020)—staying down thereafter.^26^ Results are similar when we exclude from the sample counties that adopted safer-at-home orders or non-essential business closures early in the pandemic (Fig. 5 in the Appendix).^27^

Another concern regarding identification refers to the start of social distancing, as well as the observance of business closures and safer-at-home orders. If residents in early-adopting counties were already practicing self-distancing prior to the adoption of an NPI, its estimated effectiveness in curtailing deaths would be biased upward. (The opposite would be the case if, instead, that was predominantly the case among residents in counties that were late adopters of NPIs—namely, the estimated impact of the NPI would be biased downward.) In addition, it is important for the orders to have been observed by the public similarly across counties. If orders were observed differentially in counties that were early versus late adopters, the estimated impact of the NPI could also be biased. Fortunately, both concerns have been addressed by recent research (e.g., Alexander and Karger 2020) showing that county-level measures of mobility declined sharply the day after stay-at-home orders went into effect, but not prior to their implementation.

## Mechanisms

According to the Centers for Disease Control and Prevention, the COVID-19 virus is primarily transmitted between people through respiratory droplets emitted during coughing or sneezing and through fomites in the environment around infected persons.^28^ By reducing close contact between individuals, the adoption of NPIs may slow down virus transmission and consequently, curtail deaths. In the absence of vaccines and reliable tracking systems, NPIs have also been invoked to flatten the pandemic curve by lowering the demands on public healthcare services, allowing for COVID-19 patients to be properly treated (Ferguson et al. 2020). As such, NPIs could have helped lower COVID-19 mortality directly by reducing contagion, as well as indirectly by preventing bottlenecks in the healthcare system.

In an attempt to sort out these two channels, we look first at COVID-19 infections. If the effectiveness of the NPIs did not stem from reducing contagion but, rather, from avoiding an overwhelmed healthcare system, we should not observe a reduction in the infection rate. As displayed in columns (1) through (3) of Table 5, adopting one of the two types of NPIs under examination 1 day before infections double would have reduced infections by roughly 1%, regardless of whether we use all counties, exclude New York, or exclude the entire Northeast region.

**Table 5:** Exploring main mechanism—stemming contagion and/or an overwhelmed healthcare system

Outcome	COVID-19 infections per 100,000			Non-COVID-19 deaths per 100,000		
Column	(1)	(2)	(3)	(4)	(5)	(6)
Sample	All counties	Excluding NY	Excluding NE region	All counties	Excluding NY	Excluding NE region
*Post*_*ct*_ × *NPI Speed*_*c*_	−0.0172***	−0.0166***	−0.0130***	0.0119	0.0122	0.0070
	(0.0024)	(0.0024)	(0.0023)	(0.0223)	(0.0230)	(0.0214)
State-level tests per 100,000	0.0029***	0.0029***	0.0014***	−0.0000	−0.0000	−0.0000*
	(0.0005)	(0.0005)	(0.0005)	(0.0000)	(0.0000)	(0.0000)
*Post*_*ct*_ × *Other NPI speed*_*c*_	−0.0241***	−0.0232***	−0.0203***	−0.0856	−0.0826	−0.0449
	(0.0018)	(0.0018)	(0.0017)	(0.0533)	(0.0536)	(0.0434)
Observations	215,073	211,071	200,721	561	550	462
*R*-squared	0.123	0.110	0.093	0.908	0.904	0.930
Dependent variable mean	1.648	1.582	1.445	25.160	25.355	25.383

Notes: *** *p*<0.01, ** *p*<0.05, * *p*<0.1. All regressions include a constant term, date, and county fixed effects. This table reports estimates using specification (3) from Table 2 that predicts daily COVID-19 deaths occurring between February 15, 2020, and April 23, 2020, and controls for state-level testing and other NPI adoption speed

We then look at how the speed of NPI adoption might have affected non-COVID mortality. If NPIs primarily helped curtail deaths by preventing the healthcare system from becoming overwhelmed, non-COVID mortality rates should also be lower in those regions where NPIs were adopted early, as non-COVID patients could still be treated. Based on the results in columns (4) through (6) in Table 5, the estimated coefficients are not statistically different from zero. In other words, the response speed does not significantly alter non-COVID deaths, as one would expect if preventing congestion of the healthcare system was a primary channel for the reduction in COVID-19 mortality following NPI adoption. Thus, our estimates suggest that excess deaths from non-COVID causes observed in other studies (e.g., Woolf et al. 2020) during the period of our study were not the result of NPI adoption speed.

In sum, the results in Table 5 point to the efficacy of NPIs in reducing COVID-19 mortality by curtailing contagion and the spread of the virus. These findings prove robust when we further distinguish between business closures and safer-at-home orders, as displayed in Table 10 of the Appendix.^29^

## Heterogeneous impacts

To conclude, we examine if the relative adoption speed of NPIs impacted counties differently based on other traits associated with either their adoption or the disproportionate toll of the virus. Attitudes toward the efficacy of NPIs have been partisan, with Republican governors and mayors being more reluctant to implement NPIs (Funk and Tyson 2020). It is possible that residents in majority-Republican counties with NPIs in place may be less compliant with healthcare guidelines and recommendations. Given the role of political partisan ideology in NPI adoption (Dave et al. 2020; Gupta et al. 2020), we examine the differential impact that the speed of NPI adoption had in Republican counties—measured as those with a Republican vote share above 50% in the 2016 presidential election.^30^ As can be seen in column (1) of Table 6, adopting an NPI 1 day before infections double lowers COVID-19 mortality in majority-Republican counties by approximately 1.5%, whereas the reduction in other counties reaches 4.4%. In other words, speeding up the implementation of NPIs in Republican counties would lower COVID-19 mortality by a third of the amount it would in non-Republican counties.^31^ Why? Perhaps, as pointed out by Engle et al. (2020) and Brodeur et al. (2020), counties with lower share of votes for Republicans comply more with NPIs, in which case, speeding up their implementation does not have the same bite as in more liberal counties. The descriptive statistics in Table 1 show that, according to our NPI speed measure, Republican counties were more likely to be early adopters, largely on account of the late outbreak of the pandemic in these counties. Thus, our findings suggest that, despite the advantage of learning from the experience of counties where the virus spread earlier, NPIs were less effective in these counties.

**Table 6:** Heterogeneous effects of NPI speed on COVID-19 deaths per 100,000 residents

Column	(1)	(2)	(3)	(4)	(5)	(6)	(7)
County characteristic (*CC*)	Majority republican	% over 65	% uninsured	% unemployed	% below FPL	Comorbidity index	Population density
*Post*_*ct*_ × *NPI Speed*_*c*_	−0.0031***	−0.0025***	−0.0014***	−0.0012***	−0.0011***	−0.0013***	−0.0009***
	(0.0006)	(0.0006)	(0.0003)	(0.0003)	(0.0004)	(0.0002)	(0.0003)
*Post*_*ct*_ × *NPI Speed*_*c*_ × *CC*	0.0020***	0.0001**	0.00002	−0.0001	−0.00001	−0.00004	−0.000005
	(0.0006)	(0.0000)	(0.00005)	(0.0002)	(0.00004)	(0.0001)	(0.000004)
State-level tests per 100,000	0.0003***	0.0003***	0.0003***	0.0003***	0.0003***	0.0003***	0.0002***
	(0.0000)	(0.0000)	(0.0000)	(0.0000)	(0.0000)	(0.0000)	(0.0000)
*Post*_*ct*_ × *Other NPI speed*_*c*_	−0.0016***	−0.0016***	−0.0016***	−0.0016***	−0.0016***	−0.0016***	−0.0015***
	(0.0001)	(0.0001)	(0.0001)	(0.0001)	(0.0001)	(0.0001)	(0.0001)
Observations	213,141	215,073	215,004	215,004	215,004	215,073	214,935
*R*-squared	0.084	0.084	0.084	0.084	0.084	0.084	0.089
Dependent variable mean	0.071	0.070	0.070	0.070	0.070	0.070	0.070

Notes: *** *p*<0.01, ** *p*<0.05, * *p*<0.1. All regressions include a constant term, date, and county fixed effects. Standard errors are in parentheses and clustered at the county level. Observations vary across specifications due to missing data. Column (1) uses a dummy variable to indicate counties where the Republican vote share in the 2016 presidential election exceeded 50%. We rely on information from 3089 counties because 28 were missing information on election returns. Columns (3), (4), and (5) use information from 3116 counties because one county was missing information on the number of residents without health insurance, unemployed, or living below the federal poverty level. In column (7), we use information from 3115 counties because two counties were missing land area information required to calculate the number of residents per square mile

**Table 11:** Effects of NPI speed on COVID-19 deaths per 100,000 residents by republican vote share

County characteristic (*CC*)	Republican vote share
*Post*_*ct*_ × *NPI Speed*_*c*_	−0.0012***
	(0.0003)
*Post*_*ct*_ × *NPI Speed*_*c*_ × *CC*_*Under* 40%_	−0.0037***
	(0.0013)
*Post*_*ct*_ × *NPI Speed*_*c*_ × *CC*_*Over* 60%_	0.0001
	(0.0003)
State-level tests per 100,000	0.0003***
	(0.0000)
*Post*_*ct*_ × *Other NPI speed*_*c*_	−0.0012***
	(0.0001)
Observations	213,141
*R*-squared	0.089
Dependent variable mean	0.071

Notes: *** *p*<0.01, ** *p*<0.05, * *p*<0.1. All regressions include a constant term, date, and county fixed effects. Standard errors are in parentheses and clustered at the county level. This model uses dummy variables to indicate the county’s Republican vote share in the 2016 presidential election where the reference category is counties at the margin (40–60% Trump vote shares). We rely on information from 3089 counties because 28 were missing information on election returns

Finally, we consider how NPI effectiveness varied according to various county-level traits known to be correlated to COVID-19 mortality. To that end, we first explore if the adoption speed of NPIs benefits localities with a higher share of individuals age 65 and older. As shown in column (2) of Table 6, we do not find that to be the case. This could be because most COVID-related elderly fatalities over our study period were in nursing homes, where safer-at-home orders and non-essential business closures might not have been as effective in reducing contagion. Residents of nursing homes would require other measures limiting their potential exposure to the virus, such as restrictions of visitors or the safe distancing of residents from each other.

We then repeat the same exercise using other county-level traits reflective of the share of the population that lack health insurance, are unemployed, or live below the federal poverty line. In addition, we explore if speeding up the implementation of NPIs has any differential impact in counties with higher comorbidity indexes and population density. As seen in columns (3) through (7) in Table 6, speeding up the implementation of NPIs does not differentially alter COVID-19 mortality in counties with different values of the abovementioned traits. To some degree, this is not surprising given their aggregated nature—as such, their non-significance should be interpreted with caution.

## Summary and conclusions

The rapid spread of the COVID-19 pandemic took the world by surprise. In the absence of a vaccine in the early stages of the pandemic, several countries opted for the adoption of non-pharmaceutical interventions (NPIs) to halt the devastating impact of the pandemic on human life. The United States was no different in that regard, even though the response has been more fragmented and piecemeal. Prior research has shown the effectiveness of NPIs in curtailing deaths in the United States, Europe, and Asia. Our focus is on the importance of their timeliness, the mechanisms behind it, and the heterogeneity of any effectiveness based on county political ideology and population susceptibility to the virus.

Using county-level data on COVID-19 mortality and infections, along with county-level information on the adoption of safer-at-home orders and non-essential business closures, we examine how the adoption speed of NPIs affected COVID-19 mortality. We find that moving up the implementation date of NPIs by 1 day before infections double lowers the COVID-19 death rate by 1.9%. The effectiveness of acting early is similar for both safer-at-home orders and business closures. An event study addresses concerns regarding the endogeneity of NPI adoption, and robustness checks show that our results persist when introducing controls for testing, the speed of adopting other NPIs, and mobility, as well as altering the definition of adoption speed, applying population weights, and considering different geographic scopes. Finally, we confirm how the relevance of responding early stems from the ability to slow contagion, which likely prevented the overburdening of the healthcare system. We also find that NPI adoption speed was less effective in Republican counties—a possible by-product of skepticism and reluctance to apply or fully comply with NPIs. In contrast, NPIs appear similarly effective, and their speed of implementation equally as relevant, in counties with distinct degrees of vulnerability to the disease, as captured by an index of comorbidity and the share of residents without health insurance, unemployed, or living in poverty.

While we await the vaccination of millions of people and a cure for those infected, NPIs remain the primary means to curtail COVID-19 infections and deaths. Gaining a better understanding of their timeliness and the importance of responding early is essential, especially in the foreseeable occurrence of additional waves.

## Appendix

**Table 7:** Alternative lags to estimate the impact of NPI speed on COVID-19 deaths per 100,000 residents

Model specification	Baseline	Control for testing	Control for other NPI speed
Column	(1)	(2)	(3)
Panel A: 1-day lag			
*Post*_*ct*_ × *NPI speed*_*c*_	−0.0016***	−0.0018***	−0.0013***
	(0.0002)	(0.0002)	(0.0002)
Observations	215,073	215,073	215,073
*R*-squared	0.083	0.088	0.088
Dependent variable mean	0.070	0.070	0.070
Panel B: 5-day lag			
*Post*_*ct*_ × *NPI speed*_*c*_	−0.0018***	−0.0019***	−0.0014***
	(0.0002)	(0.0002)	(0.0002)
Observations	215,073	215,073	215,073
*R*-squared	0.083	0.088	0.089
Dependent variable mean	0.070	0.070	0.070
Panel C: 10-day lag			
*Post*_*ct*_ × *NPI speed*_*c*_	−0.0018***	−0.0020***	−0.0013***
	(0.0002)	(0.0002)	(0.0002)
Observations	215,073	215,073	215,073
*R*-squared	0.083	0.088	0.089
Dependent variable mean	0.070	0.070	0.070

Notes: *** *p*<0.01, ** *p*<0.05, * *p*<0.1. All regressions include a constant term, date, and county fixed effects. Standard errors are in parentheses and clustered at the county level. The specification in column (1) only includes date and county fixed effects, column (2) controls for state-level testing, and column (3) further controls for the speed of adopting other NPIs

**Table 8:** Alternative models estimating the impact of NPI speed on COVID-19 deaths per 100,000 residents

Model specification	Baseline	Control for testing	Control for other NPI speed
Column	(1)	(2)	(3)
Panel A: Restricted sample			
*Post*_*ct*_ × *NPI speed*_*c*_	−0.0022***	−0.0023***	−0.0013***
	(0.0002)	(0.0002)	(0.0002)
Observations	141,480	141,480	141,480
*R*-squared	0.119	0.125	0.127
Dependent variable mean	0.100	0.100	0.100
Panel B: Restricted sample and disaggregated NPI speeds			
*Post*_*ct*_ × *BC Speed*_*c*_	−0.0019***	−0.0014***	−0.0007***
	(0.0002)	(0.0002)	(0.0002)
*Post*_*ct*_ × *SAH Speed*_*c*_	−0.0009***	−0.0012***	−0.0009***
	(0.0002)	(0.0002)	(0.0002)
Observations	141,480	141,480	141,480
*R*-squared	0.120	0.126	0.127
Dependent variable mean	0.100	0.100	0.100
Panel C: Full sample and mutually exclusive NPI speeds			
*Post*_*ct*_ × *Only BC Speed*_*c*_	−0.0021***	−0.0012***	−0.0004
	(0.0002)	(0.0002)	(0.0002)
*Post*_*ct*_ × *Only SAH Speed*_*c*_	−0.0019***	−0.0011**	−0.0003
	(0.0004)	(0.0004)	(0.0004)
*Post*_*ct*_ × *BC and SAH Speed*_*c*_	−0.0022***	−0.0022***	−0.0014***
	(0.0002)	(0.0002)	(0.0002)
Observations	215,073	215,073	215,073
*R*-squared	0.084	0.088	0.089
Dependent variable mean	0.070	0.070	0.070

Notes: *** *p*<0.01, ** *p*<0.05, * *p*<0.1. All regressions include a constant term, date, and county fixed effects. Standard errors are in parentheses and clustered at the county level. Panels A and B predict daily COVID-19 deaths occurring between March 1, 2020, and April 23, 2020, for the 2260 counties with mobility data. Panel C predicts COVID-19 deaths occurring between February 15, 2020, and April 23, 2020, for the 3117 counties in our full sample and differs from our main specifications in two ways: (1) non-essential business closure and safer-at-home order speeds are now mutually exclusive and (2) a separate measure capturing the speed of joint adoption is included. Column (2) controls for state-level testing, and column (3) further controls for the speed of adopting other NPIs

**Table 9:** Robustness checks—impact of NPI speed on COVID-19 deaths per 100,000 residents

Robustness check	Alternative contagion threshold		Alternative weighting	Alternative samples		
Column	(1)	(2)	(3)	(4)	(5)	(6)
Model specification	Pre-NPI national average	Pre-NPI county average	Population weighted	Excluding NY	Excluding NE region	Only the NE region
*Post*_*ct*_ × *BC Speed*_*c*_	−0.0008***	0.0009*	−0.0003	−0.0005***	−0.0005**	−0.0048***
	(0.0002)	(0.0005)	(0.0006)	(0.0002)	(0.0002)	(0.0015)
*Post*_*ct*_ × *SAH Speed*_*c*_	−0.0006***	−0.0028***	−0.0030***	−0.0008***	−0.0005***	−0.0101***
	(0.0002)	(0.0006)	(0.0008)	(0.0002)	(0.0002)	(0.0022)
State-level tests per 100,000	0.0003***	0.0003***	0.0006***	0.0003***	0.0002***	0.0001*
	(0.0000)	(0.0000)	(0.0001)	(0.0000)	(0.0000)	(0.0001)
*Post*_*ct*_ × *Other NPI Speed*_*c*_	−0.0005***	−0.0028***	−0.0028***	−0.0010***	−0.0009***	−0.0028**
	(0.0001)	(0.0002)	(0.0004)	(0.0001)	(0.0001)	(0.0014)
Observations	215,073	215,073	215,073	211,071	200,721	14,352
*R*-squared	0.088	0.089	0.274	0.085	0.072	0.253
Dependent variable mean	0.070	0.070	0.155	0.068	0.060	0.207

Notes: *** *p*<0.01, ** *p*<0.05, * *p*<0.1. All regressions include a constant term, date, and county fixed effects. This table reports estimates using specification (3) from Table 2 that predicts daily COVID-19 deaths occurring between February 15, 2020, and April 23, 2020. In columns (1) and (2), we alter the definition of contagion we used to measure the speed of NPI adoption. Specifically, we replace our original contagion threshold, which reflected the first day-to-day doubling of infections per capita in a given county to (1) the first day infections per capita exceeded the national average from January 21, 2020, to March 7, 2020, and (2) the first day infections per capita exceeded the overall county average prior to any NPI adoption, the results of which are found in columns (1) and (2), respectively. In column (3), we apply population weights to derive nationally representative estimates. In columns (4), (5), and (6), we experiment with using alternative samples. In column (4), we exclude New York from the analysis. In column (5), we exclude the entire Northeast region, which consists of Connecticut, Maine, Massachusetts, New Hampshire, Rhode Island, Vermont, New Jersey, New York, and Pennsylvania. In column (6), we focus exclusively on the Northeast region

**Table 10:** Exploring main mechanism—stemming contagion and/or an overwhelmed healthcare system

Outcome	COVID-19 infections per 100,000			Non-COVID-19 deaths per 100,000		
Column	(1)	(2)	(3)	(4)	(5)	(6)
Sample	All counties	Excluding NY	Excluding NE region	All counties	Excluding NY	Excluding NE region
*Post*_*ct*_ × *BC Speed*_*c*_	−0.0074***	−0.0080***	−0.0097***	0.0250	0.0251	0.0434*
	(0.0029)	(0.0029)	(0.0030)	(0.0334)	(0.0337)	(0.0232)
*Post*_*ct*_ × *SAH Speed*_*c*_	−0.0110***	−0.0104***	−0.0067***	−0.0300	−0.0302	−0.0373
	(0.0023)	(0.0023)	(0.0022)	(0.0359)	(0.0362)	(0.0239)
State-level tests per 100,000	0.0028***	0.0028***	0.0013***	−0.0000	−0.0000	−0.0000*
	(0.0005)	(0.0005)	(0.0005)	(0.0000)	(0.0000)	(0.0000)
*Post*_*ct*_ × *Other NPI Speed*_*c*_	−0.0223***	−0.0212***	−0.0179***	−0.0790	−0.0757	−0.0438
	(0.0018)	(0.0018)	(0.0017)	(0.0517)	(0.0518)	(0.0452)
Observations	215,073	211,071	200,721	561	550	462
*R*-squared	0.123	0.111	0.093	0.908	0.905	0.930
Dependent variable mean	1.648	1.582	1.445	25.160	25.355	25.383

Notes: *** *p*<0.01, ** *p*<0.05, * *p*<0.1. All regressions include a constant term, date, and county fixed effects. This table reports estimates using specification (3) from Table 3 that predicts daily COVID-19 deaths occurring between February 15, 2020, and April 23, 2020, and controls for state-level testing and the speed of adopting other NPIs

## Footnotes

1 Other studies examine socioeconomic factors, such as income, influencing compliance with some of these self-protective behaviors (e.g., Papageorge et al. 2021).

2 Relatedly, other authors focus on assessing the factors conditioning compliance with safer-at-home orders, such as trust and risk perception using mobility data (e.g., Bargain and Ulugbek 2020; Brodeur et al. 2020; Brzezinski et al. 2020; Engle et al. 2020).

3 We follow Amuedo-Dorantes et al. (2020) who used a similar measure to study the effectiveness of NPIs in Spain and Correia et al. (2020) who studied the effect of NPIs during the 1918 influenza.

4 We conduct this analysis at the state level because county-level non-COVID deaths are not consistently available for the period under consideration in our study.

5 Retrieved on July 20, 2020, from https://github.com/CSSEGISandData/COVID-19. Using documentation provided by Johns Hopkins, we identified 34 counties that required consolidation to align with the level at which COVID-19 cases and deaths were reported. This affected five counties in New York (New York City’s five boroughs were originally grouped under New York county), two counties in Massachusetts (Dukes and Nantucket), Rhode Island’s five counties, and 22 counties in Utah. In each of these instances, we aggregate population counts and apply population weights to approximate aggregated characteristics. These consolidations bring our county total to 3117 down from 3142. Cases and deaths not assigned to a geographic area are excluded from our analysis.

6 The state of Alaska lifted its safer-at-home and non-essential business closure orders on April 24, 2020.

7 Retrieved on July 27, 2020, from https://covidtracking.com/data/download. We make use of state-level data because data on testing is not consistently available at the county level for the period under consideration.

8 Retrieved on July 20, 2020, from https://data.cdc.gov/NCHS/Weekly-counts-of-death-by-jurisdiction-and-cause-o/u6jv-9ijr. Data on non-COVID deaths is not available at the county level for the period under consideration.

9 Retrieved on July 20, 2020, from https://ce.naco.org/.

10 Retrieved on July 20, 2020 from https://tinyurl.com/statepolicies.

11 Activities and businesses deemed “essential” vary by county and state.

12 Retrieved on December 21, 2020, from https://healthdata.gov/dataset/covid-19-state-and-county-policy-orders.

13 Retrieved on July 20, 2020, from https://github.com/descarteslabs/DL-COVID-19.

14 Retrieved on July 20, 2020, from https://dataverse.harvard.edu/dataverse/medsl_president.

15 Retrieved from https://www.cms.gov/Research-Statistics-Data-and-Systems/Statistics-Trends-and-Reports/Chronic-Conditions/CC_Main. List of high-risk conditions made available by the CDC: https://www.cdc.gov/coronavirus/2019-ncov/hcp/underlying-conditions.html.

16 Retrieved from https://www.census.gov/library/publications/2011/compendia/usa-counties-2011.html#LND.

17 We experiment with different contagion thresholds in robustness checks outlined later in the paper. Results prove consistent throughout.

18 This approach aligns with that used in Correia et al. (2020).

19 We would have preferred to control for county-level testing, but consistent data are not publicly available.

20 Recall that our baseline specification includes a 14-day lag. We also experiment with alternate lags of 1, 5, and 10 days. As shown in Table 7 of the Appendix, our results prove robust to alternative incubation periods.

21 Computed as: (*β* ∗ *ΔX*) ∗ 100/*μ*_*DV*_, where: Δ*X*=1 and *μ*_*DV*_=0.070.

22 The effects of these two NPIs are not statistically different from one another: F(1, 3117) = 1.03, *p*=0.309.

23 The Northeast region includes Connecticut, Maine, Massachusetts, New Hampshire, Rhode Island, Vermont, New Jersey, New York, and Pennsylvania.

24 National infection rates between January 1 and March 7, 2020, averaged 7.30 infections per 100,000. The pre-NPI county average infection rate was 1.85 infections per 100,000.

25 Results are similar when we further separate non-essential business closures and safer-at-home orders, as shown in Table 9 of the Appendix. The sole exception is the estimated coefficient on the speed of non-essential business closures in column (2), which is positive and marginally significant at the 10% level.

26 Prior epidemiological studies have documented that, early in the pandemic, the median time between initial COVID-19 symptoms and death was 19 days, roughly a 3-week period (Zhou et al. 2020), which when added to the 1–2-week incubation period, takes us to the observed impact 4–5 weeks after the NPI adoption.

27 We also conduct the analysis using confirmed cases, despite mismeasurement concerns. Results, available from the authors, suggest an earlier effectiveness of NPIs in reducing confirmed cases when compared to deaths.

28 See: https://www.cdc.gov/coronavirus/2019-ncov/faq.html#Spread.

29 While the estimated coefficient on NPI speed is positive in column (6) of Table 10, it is only marginally significant at the 10% level.

30 In turn, other studies examine how the ability to control the pandemic impacted political support and the 2020 elections (e.g., Baccini et al. 2021).

31 We also compare counties with Republican vote shares in the upper (more than 60%) and lower (less than 40%) ends of the distribution with counties at the margin (Lee et al. 2004). As can be seen in Table 11 in the Appendix, relative to counties where 40 to 60% of residents voted Republican, those at the lower end of the distribution—that is, those with the lowest share of Republican votes—benefited the most from early NPI adoption.
